# Modelling Random Coincidences in Positron Emission Tomography by Using Singles and Prompts: A Comparison Study

**DOI:** 10.1371/journal.pone.0162096

**Published:** 2016-09-07

**Authors:** Josep F. Oliver, M. Rafecas

**Affiliations:** Instituto de Física Corpuscular (IFIC - UV/CSIC), Valencia, Spain; University of Chicago, UNITED STATES

## Abstract

Random coincidences degrade the image in Positron Emission Tomography, PET. To compensate for their degradation effects, the rate of random coincidences should be estimated. Under certain circumstances, current estimation methods fail to provide accurate results. We propose a novel method, “Singles–Prompts” (SP), that includes the information conveyed by prompt coincidences and models the pile–up. The SP method has the same structure than the well-known “Singles Rate” (SR) approach. Hence, SP can straightforwardly replace SR. In this work, the SP method has been extensively assessed and compared to two conventional methods, SR and the delayed window (DW) method, in a preclinical PET scenario using Monte–Carlo simulations. SP offers accurate estimates for the randoms rates, while SR and DW tend to overestimate the rates (∼10%, and 5%, respectively). With pile-up, the SP method is more robust than SR (but less than DW). At the image level, the contrast is overestimated in SR-corrected images, +16%, while SP produces the correct value. Spill–over is slightly reduced using SP instead of SR. The DW images values are similar to those of SP except for low-statistic scenarios, where DW behaves as if randoms were not compensated for. In particular, the contrast is reduced, −16%. In general, the better estimations of SP translate into better image quality.

## Introduction

Positron emission tomography (PET) is based on the detection, in coincidence, of two photons created upon the annihilation of a positron. Due to the finite time resolution of PET devices, a coincidence event is recorded when the two annihilation photons are detected within a time coincidence window (TCW). Within this scheme it is unavoidable that two uncorrelated photons might be detected sufficiently close in time to be mistakenly identified as a coincidence. This constitutes an *accidental* coincidence, also called *random* coincidence (or just *random*). Randoms are one of the main sources of image degradation in PET, since they introduce noise and hamper quantification. The negative effects of randoms can be partially compensated for, either by pre–correcting the data prior to reconstruction [1] or within the reconstruction process [2]. Both approaches require a reliable estimate of the number of randoms in each line–of–response (LoR). For quantitative PET, accurate randoms estimates are imperative.

Two estimation approaches are usually employed: the “Delayed Window” (DW) method [1, 3] and the “Singles Rate” (SR) method [1, 3, 4]. The DW method duplicates the stream of events and the detection times are delayed for a time much larger than the time resolution of the scanner. Coincidences between the original and the delayed copy are extracted using the same sorter. The DW method relies on the fact that the correlations between the original and delayed copy are broken; therefore, any coincidence between the two is necessarily a random. The prompts rate obtained for each LoR defined by the detectors *ij*, $R_{ij}^{\text{DW}}$, constitutes the DW estimate. The SR method uses the singles count rates of two detectors to infer the randoms rate in the corresponding LoR. It is based on the well–known formula:

$$\begin{array}{c} R_{ij}^{\text{SR}}=2\tau S_{i}S_{j}, \end{array}$$ (1)

being $R_{ij}^{\text{SR}}$ the estimated randoms rate for the LoR defined by the detectors *ij* and *τ* the value of the TCW. This method relies on the assumption that the singles rates are large compared with the trues rates.

Several studies compare the performance of these two methods [5–9]. While more accurate, the DW method presents two main drawbacks: if it is directly implemented on hardware, additional circuitry is required for the delayed channel, which contributes to increase the system dead–time. Increased dead–time can be avoided if it is implemented post–acquisition in software [9]. Even in this case, DW estimates are affected by higher levels of statistical noise than SR estimations because the latter are based on counting singles while the former are based on counting coincidences [7]. Because of this, the SR method is sometimes preferred. The results reported in previous works [8–10] indicate that the SR method systematically overestimates the correct randoms rate. In [10] we proposed an (iterative) extension of the SR method that provides estimates compatible with the correct value. Yet, this extension is not able to provide accurate estimates for scenarios in which the count rate is so high that the probability of finding more than one event inside the TCW cannot be neglected. As the activity increases, so does the rate of events detected, and the higher the rate of events, the lower the probability of finding only one event inside the TCW opened by another event. We will refer to this effect as *pile–up* [10].

The aim of this paper is to extend the conventional SR approach by exploiting the information contained in the singles *and* prompts rates. The novel method, termed the “Singles–Prompts” (SP) method, only uses measurable data and provides the correct value for the randoms rate in one step (i.e. avoiding iterations) even for high count rate scenarios. A preliminary version of SP was introduced in [11, 12].

The SP method is applicable to any kind of scanner in which the detected photon interaction is assigned to a volume, regardless of the read-out. For block read–out schemes, the volume is a crystal element; it can also be a “detector voxel” [13]. In particular, the method applies to crystals individually read out. For concreteness, we adopt the latter approach and assume singles list–mode data. The high flexibility that this format provides has triggered a growing interest in the last years [14–18]. We have implemented a generic small animal PET scanner that provides a concrete scenario to study the performance of the methods. The scanner has been designed to provide excellent geometrical coverage, following current instrumentation trends [19–21].

To assess the performance of the methods at the data level, the true number of randoms present in the data should be known. Since this information is not available in real data, we have resorted to Monte-Carlo simulations. Quantitative assessment of the performance of the methods has been also done at image level. To this end, standard figures–of–merit (FoM) were calculated for random-compensated images of various phantoms. The dependence of the FoMs on the statistical quality of the data has been also investigated. Finally, images of the MOBY phantom [22] have been reconstructed for visual comparison.

## Methods

### Nomenclature and definitions

*Detector element* (or simply *detector*): each of the volumes which can be assigned to a detected interaction. For scanners based on pixelated or segmented crystals, like the one used in this paper, each of the small crystal units constitutes a detector. However, a detector might not correspond to a physical element; monolithic crystals could be virtually divided into imaginary subcrystals, and each virtual subcrystal would thus constitute a detector.

**Fig 1:**
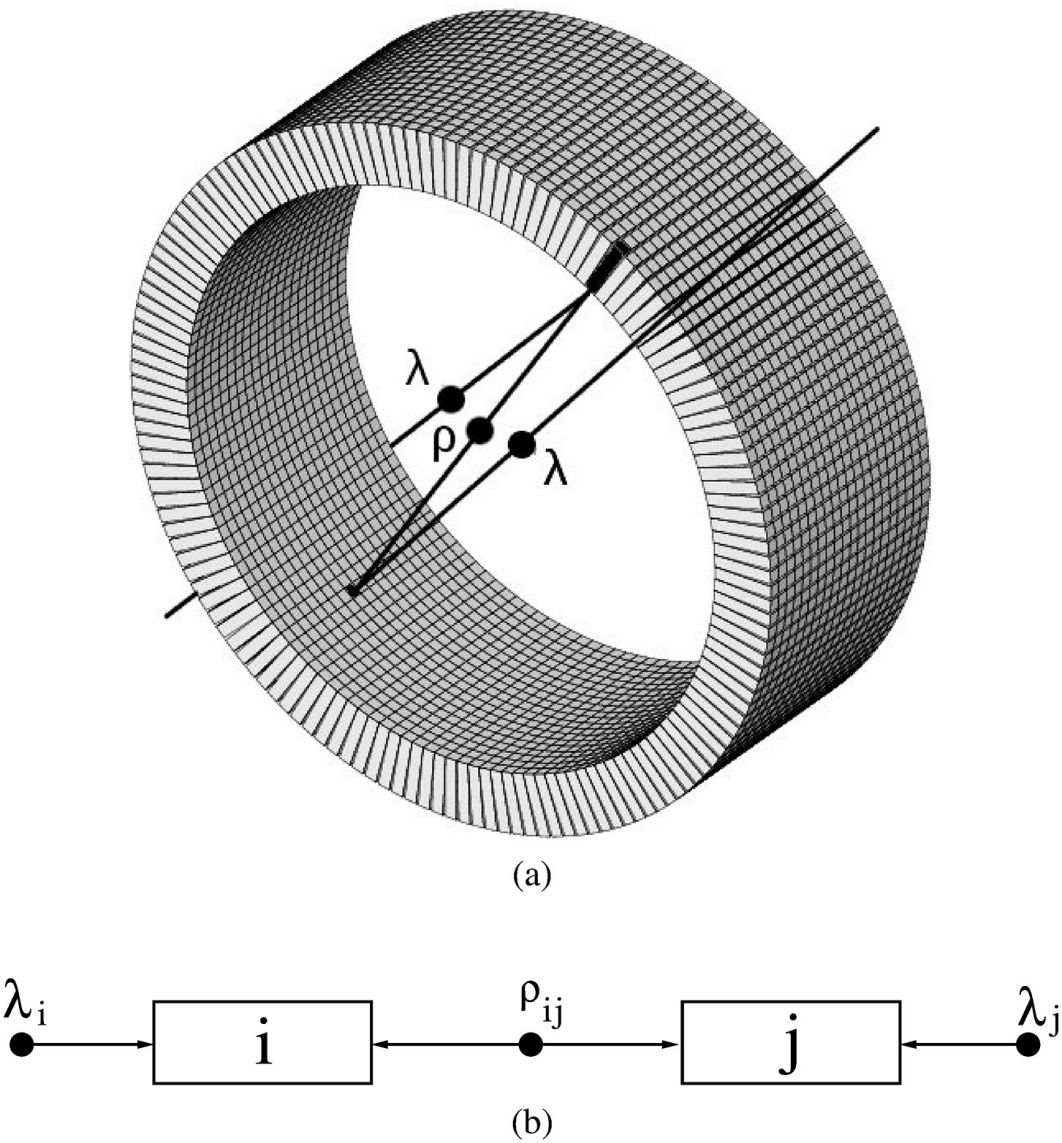
(a): Test scanner. The filled circles represent positron annihilations and the lines represent the trajectory of the resulting annihilation photons. For some annihilations, one photon of the pair is detected while the other is lost; for some other annihilations both photons are detected. (b): schematic representation of the detection model. The photons measured by the *i*^*th*^ detector can be modelled as those originating from a source of individual photons (it represents the situation where only one photon is detected), plus photons from a source of photon pairs (both photons are detected).

*Single*: Each of the individual detection events assigned to a *detector*. In this paper we use a model for the signal very similar to the one used in [23]. Each positron annihilation creates two almost back–to–back photons of 511 keV. A possibility is that only one interaction of one of the photons is detected; i.e. one annihilation gives rise to one *single*. This kind of signal is referred to as *uncorrelated*. It may also happen that both photons give rise to one *single* each. This case is termed *correlated* signal, and each of the two singles, *correlated* singles. The singles detected by each member, *i*, of a pair of *detector elements*, *ij*, can be understood as the sum of *singles* coming from an *uncorrelated* source and *singles* from a *correlated* source, Fig 1.

To a good degree of approximation, positron annihilations and subsequent photon emissions occur according to a Poisson process. Each pair of photons can be classified into one of the following outcomes: (1) no photon is detected; (2) only one photon is detected, and (3) both photons are detected. The samples obtained upon classification of Poisson-distributed samples also obey a Poisson distribution.

Therefore, the generation of individual *uncorrelated singles* as well as the generation of pairs of *correlated singles* can be properly described by Poisson distributions with expected values *λ*_*i*_ and *ρ*_*ij*_, respectively. (See Fig 1.)

Because of energy conservation, any annihilation photon (511 keV) can produce at most one energy deposition higher that 255.5 keV (i.e., 511/2 keV). Therefore, two limiting scenarios are possible depending on the value used for the low energy threshold (LET). For $E_{\text{LET}}>255.5\;\text{keV}$, only one single per photon is possible, while for $E_{\text{LET}}\leq 255.5\;\text{keV}$ the 511-keV photon can give rise to more than one single through Compton scattering. When raising $E_{\text{LET}}$, the transition between these scenarios is not abrupt. Due to energy resolution it is possible that for $E_{\text{LET}}>255.5\;\text{keV}$ some photons give rise to more than two singles. However, the probability of this kind of events will decrease as $E_{\text{LET}}$ is increased. For conventional photo–peak centered energy windows [450, 750] keV, this probability is negligible.

PET is based on the detection and identification of pairs of annihilation photons. These pairs can be identified at the hardware level using coincidence units or, in some scanners, they can be extracted post-acquisition using dedicated data processing algorithms. We will use the term *sorter* to refer to any process designed to identify the pairs of annihilation photons present in the data.

*Prompt coincidence*, or *prompt*: event made of two *singles* paired by the sorter. Prompts are further classified into *true coincidences*, or *trues*, and *random coincidences*, or *randoms*. A *random* is a *prompt* in which the two *singles* correspond to photons that were not created in the same positron annihilation (two *uncorrelated singles*). We define a *true coincidence* as the opposite, i.e. a *prompt* in which the two *singles* are due to photons created in the same positron annihilation (*correlated singles*). Note that this is not the usual definition of a “true coincidence” since our definition also includes “scatter coincidences”. Our nomenclature is justified because, as shown later, to estimate the randoms rate, our model does not require to know whether the photon underwent scattering before its detection. For $E_{\text{LET}}>255.5\;\text{keV}$, a *true* is made of two *correlated singles*, while for lower LET values this is not always the case.

In the forthcoming, *S*_*i*_ is defined as the singles rate measured by the detector *i*. *R*_*ij*_, *T*_*ij*_ and *P*_*ij*_ are the random, true and prompt rates between *i* and *j*, respectively.

### Coincidence sorting methods

**Fig 2:**
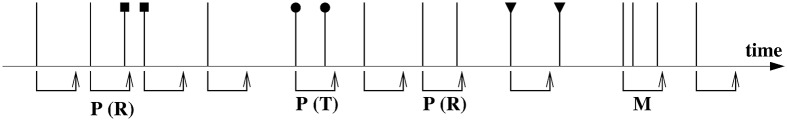
SW sorter: Schematic representation. Time is represented horizontally. Uncorrelated singles are depicted as vertical lines and pairs of correlated singles are depicted with equal symbols on their tops. The TCW is represented as a bended arrow below the data flow. Sorted prompts are tagged as P and further classified into randoms, R, and trues, T. The multiple coincidence is tagged as M.

The sorter task is to identify the pairs of annihilation photons (*correlated singles*) on which PET is based. Most sorters rely on the fact that the detection of the annihilation photons should occur almost simultaneously. For this purpose TCWs are often used. Yet, alternative approaches not directly based on TCWs are also possible [24]. In any case, many TCW–based sorters can be described by the “*single window*” model (SW) [9, 14], particularly when the sorting is implemented in hardware using logic gates [3]. For the SW sorter, only one TCW is open at a time, as opposite to the “*multiple window*” model (MW) that can have many windows simultaneously open [25, 26]. Although differences between these sorters are known to exist, the results from [27] show that for the conventional (double) coincidences, which are the only ones of interest for the present study, the outcome of both sorters is very similar. In this work, we have implemented a SW sorter. The results and conclusions should also be also valid for a MW sorter. Following the conventional prescription, the TCW value, *τ*, used in this work is twice the time resolution (FWHM) of the scanner. The SW sorter is straightforward to implement: when a single is processed and there is no open window, a new TCW is open. Then, the sorter searches for a second single within the TCW. When only one single is found inside, both singles are grouped together and constitute a prompt. If more than one single lay inside the TCW (multiple coincidence), all singles are discarded. This process is illustrated in Fig 2.

### Random estimation methods

In the Introduction we have summarized the two most extended methods. In this section, we describe the new model as well as an ideal sorter method that is introduced for comparison purposes.

#### Singles Prompt method

In a regular PET acquisition, most of the singles are uncorrelated, while the desired signal consists of pairs of correlated singles. Since uncorrelated singles outnumber correlated singles, most of the randoms will be made of two uncorrelated singles. In fact, the assumption of considering randoms made of two uncorrelated singles was shown to be a good approximation in [10]. Within the framework of this model, the rate of randoms made of uncorrelated singles in the LoR defined by the *i* and *j* detectors reads [10]:

$$\begin{array}{c} R_{ij}=2\tau \lambda_{i}\lambda_{j}e^{-2\Lambda \tau }, \end{array}$$ (2)

where Λ ≡ ∑_*i*_ *λ*_*i*_ + 1/2∑_*i*,*j*_ *ρ*_*ij*_. However, the value of *λ*_*k*_ is not available in any acquisition.

To overcome this problem, we propose a novel estimation method, SP, also based on Eq (2). SP provides the estimate in one step, takes into account the pile–up effects and utilizes only directly measurable data. The SP estimate, $R_{ij}^{\text{SP}}$, is:

$$\begin{array}{c} R_{ij}^{\text{SP}}=\frac{2\tau e^{-(\lambda +S)\tau }}{{\left(1-2\lambda \tau \right)}^{2}}\left(S_{i}-e^{(\lambda +S)\tau }\;P_{i}\right)\left(S_{j}-e^{(\lambda +S)\tau }\;P_{j}\right), \end{array}$$ (3)

where *S* = ∑_*i*_ *S*_*i*_ is the rate of singles measured by the scanner as a whole, *P*_*i*_ = ∑_*j*_ *P*_*ij*_ is the prompts rate in detector *i* and *P* = ∑_*i*_ *P*_*i*_ is twice the prompts rate detected by the scanner; *λ* corresponds to the solution of the equation:

$$\begin{array}{c} 2\tau \lambda^{2}-\lambda +S-P\;e^{(\lambda +S)\tau }=0. \end{array}$$ (4)

The derivation of Eq (3) can be found in Appendix.

Regarding the apparent complexity of Eq (3), it is worth to stress two facts:

- SP requires the same measurements as SR, i.e., *S*_*i*_, since *P*_*ij*_ is always measured.
- The mathematical complexity of SR and SP estimators is the same since Eq (3) can be expressed as:
  $$\begin{array}{c} R_{ij}^{\text{SP}}=2\bar{\tau }{\bar{S}}_{i}{\bar{S}}_{j}, \end{array}$$ (5)
  where the *effective time coincidence window*, $\bar{\tau }$, and the *effective singles count rates* ${\bar{S}}_{i}$ are given by
  $$\begin{array}{c} \bar{\tau }=\tau \;\frac{e^{-\tau (\lambda +S)}}{{\left(1-2\tau \lambda \right)}^{2}},\;{\bar{S}}_{k}=S_{k}-P_{k}\;e^{\tau (\lambda +S)}. \end{array}$$ (6)

Incidentally, the model introduced in [10] also provides the corresponding formulas for DW and SR estimations and predicts *R*^SR^ ≥ *R*^DW^ ≥ *R*^SP^ = *R*^0^, see [28].

#### Ideal method

When using Monte–Carlo simulations, it is possible to identify the true number of randoms present in each LoR; thus, the correct randoms rate, *R*^0^_*ij*_, can be extracted from simulations. Although such an *ideal* estimation method is not possible in real acquisitions, it allows us to isolate the degradation effects due to the randoms and to determine the maximum gain achievable at the image level.

### Monte–Carlo simulations

The simulation package GATE [25, 29] was used.

#### Test scanner

A small animal PET scanner based on the values reported in [30] was simulated. It consists of 20 axial rings of 148 crystals each, Fig 1. Each of the 2 × 2 × 10 *mm*^3^ LSO crystals is read–out individually. The inner diameter is 94.2 mm and the axial length is 40 mm. An energy resolution of 15% at 511 keV (FWHM) and a time resolution of 5 ns (FWHM) are implemented. In the post-simulation sorting process, we have used a TCW of *τ* = 10 *ns* and applied a 500 ns delay for the DW method. To avoid multiple coincidences, the energy window used was [450, 750] keV. The output was singles list–mode data, providing for each single: energy, time–stamp and crystal ID.

The simulated scanner provides an excellent geometrical coverage: no gaps between the crystals and a ring diameter comparable to that of [14, 31]. Note that a good coverage tends to increase the number of correlated singles, which implies that it is less justified to ignore the correlated singles when estimating the randoms rate (an approximation on which SR heavily relies).

The main purpose of the paper is to investigate the capability of the proposed method, SP, to estimate randoms detected in each LoR. To this purpose, we have implemented two types of simulations regarding the inclusion of degradation phenomena: (1) positron range, acollinearity, dead-time and attenuation media within the object were not simulated, and (2) these effects were included. As we focus on the particular influence of the randoms on the image quality, most of the simulations were of type (1). However, to estimate the impact of the various degradation effects and their intertwining, for some scenarios the aforementioned degradation effects were simulated (type 2). As an attenuation material, a water-filled phantom was considered; to include positron range and acollinearity, fluorine–18 was used as positron emitter. Regarding the dead–time, we have used a paralysable model characterized by a dead–time value of 300 ns that is applied at the level of single events.

#### Phantoms

To investigate the dependence of the estimates on the source geometry, three phantoms have been studied.

- *Point*. A dimensionless source with all the activity concentrated into a point.
- *Mouse-like*. To simulate a source distribution with the approximated extent of a mouse, we have implemented a homogeneously active cylinder of diameter *D* = 35 mm and height *H* = 70 mm.
- *Rat-like*. Similarly, we have implemented a homogeneously active cylinder of *D* = 70 mm and *H* = 140 mm.

These phantoms are centered in the field of view (FoV). The *point* source has been used to investigate the limiting scenario in which correlated singles constitute the dominant contribution. To investigate the opposite scenario, i.e. no correlated singles are present in the data, a fourth phantom has been implemented:

- *Disc*. A homogeneously active short cylinder (*D* = 70 mm and *H* = 10 mm) was placed at 70 mm of the scanner centre and with its symmetry axis coincident with the scanner axis. Due to its placement outside the scanner, this phantom cannot produce correlated singles. Hence, all the methods should provide the correct estimation except for possible deviations due to pile–up. Therefore, this phantom allows us to focus on the latter effect.

To perform a quantitative study of the quality of the reconstructed images, a fifth phantom has been implemented:

- *Image Quality (IQ) phantom*. IQ is a homogeneously active cylinder (*D* = 48 mm and *H* = 140 mm) with two inner cavities, each being a rod of diameter 16 mm and height 50 mm. One rod was filled with a high activity concentration while the other was empty. The phantom was centred in the FoV.

To investigate the role of the source activity, a wide range spanning from 0.001 mCi to 3 mCi ([37 kBq, 111 MBq]) has been considered for the disc, point, mouse and rat phantoms. For the IQ phantom, the total activity was 1.5 mCi (55.5 MBq). One cavity was filled with an activity concentration four times higher than the background while the other was left empty. Following standard optimization procedures, the total activity was set to the NECR peak. The acquisition time was set according to the statistical requirements for each study. Without loss of generality, the activity was constant during the acquisition time. For a qualitative assessment, the MOBY phantom has been simulated, positioned inside the scanner with the mouse thorax within the FoV.

### Data Analysis

The performance of the three methods has been investigated at the data and the image level, as described below.

#### Assessment of the estimators direct output

Each pair of detectors provides a realization of the formula *R*^mth^_*ij*_, where mth stands for “method” and can take the values {DW, SR, SP, 0 (ideal)}.

For each simulation, we have computed the *total random rate*, defined as

$$\begin{array}{c} R^{\text{mth}}=\sum_{i}\sum_{j>i}R_{ij}^{\text{mth}}. \end{array}$$ (7)

The reason for using *R*^mth^ instead of $R_{ij}^{\text{mth}}$ is two folded. First, *R*^mth^ is an extensive magnitude associated with the scanner as a whole. Second, since *R*^mth^ is composed of a large sum of realizations, *R*^mth^ is less affected by statistical fluctuations between simulations than each individual $R_{ij}^{\text{mth}}$. Yet, some variability between simulations is unavoidable. Therefore, we have performed as many simulations as necessary to determine *E*[*R*^mth^/*R*^0^] with an statistical error (taken as one standard deviation) below 1%.

If we perform several acquisitions under exactly the same conditions, the values of the estimated randoms rate will spread around the mean, *E*[*R*^mth^]. Eventually, this statistical dispersion becomes an additional source of noise. Therefore, any acceptable method should provide not only an accurate estimation in average but also a low dispersion, i.e. a low variance. The variance of the SR method is known to be smaller than that of the DW method [32]. It is so small that, in general, its contribution to the noise is neglected. When calculating the NECR, the variance associated to each method is taken into account. For this purpose, we have computed the Fano factor for each method. The Fano factor is defined as the ratio between the variance and the average, *F* = *σ*^2^/*μ* [4]. Neglecting the variance of the SR method amounts to assume *F*^SR^ = 0, while for the DW method, the coefficient usually used in the NECR implies *F*^DW^ = 1, [7, 32–35]. This value reflects the fact that the DW method is based on obtaining coincidences, which is (approximately) a Poisson process.

For concreteness, we have computed the Fano factor for the IQ phantom, and we have investigated its dependence on the activity. Finally, the NECR curve for the IQ phantom has been obtained. The NECR is a metric that takes into account the statistical noise introduced by scatter and random coincidences [33]. It is considered to be a surrogate indicator of the final image quality since it provides an estimation of the quality of the measured data. To compute NECR we have used

$$\begin{array}{c} NECR=\frac{{\left(P-R^{\text{mth}}\right)}^{2}}{P-R^{\text{mth}}+\left(1+F^{\text{mth}}\right)R_{\text{pht}}^{\text{mth}}}, \end{array}$$ (8)

where *P* − *R*^mth^ is the rate of true coincidences as estimated by each method, *F*^mth^ is the Fano factor and $R_{\text{pht}}^{\text{mth}}$ is the estimated randoms rate as obtained when only the LoRs that pass through the phantom are considered [32, 36]. Other authors may adopt different definitions for $R_{\text{pht}}^{\text{mth}}$ [7, 33–35, 37]. Since we used the NECR to compare the performance of the methods, the actual definition used is not relevant as far as the same is used for all the methods. The peaks of the NECR curves have been also used to provide an estimation of the optimal working activity. It must be stressed that a NECR curve is tied to a particular scanner. In addition, the NECR peak must not be regarded as the exact value of the optimal working activity but as a reasonable estimation [36].

When compensating for randoms, it is also relevant to know their relative contribution to the measured coincidences. The correct randoms fraction, RF, was obtained from the ideal sorter.

#### Image quality assessment

It might happen that an estimation method provides an accurate value of the total number of randoms but also a very poor estimation of the number of randoms present in each LoR. Such estimates would constitute an extra source of image degradation. Therefore, it is also important to assess the performance of the methods by comparing the quality of the random–compensated images. To this end, we have reconstructed images using the three estimation methods. For reference, images using the ideal method have been also obtained. The algorithm used for the reconstruction was the gold-standard ML–EM [38], which is based on the iterative equation:

$$\begin{array}{c} f_{v}^{\left(k+1\right)}=\frac{f_{v}^{\left(k\right)}}{\sum_{l}A_{lv}}\sum_{l}\frac{y_{l}}{q_{l}^{\left(k\right)}}A_{lv}, \end{array}$$ (9)

where *y*_*l*_ is the number of prompts in LoR *l*, *A*_*lv*_ is an element of the system matrix, and $f_{v}^{\left(k\right)}$ is the reconstructed intensity inside voxel *v* for the *k*^*th*^ iteration. Finally, *q*_*l*_ is the expected value of the number of counts in *l*, which can be decomposed into the usual contribution plus the contribution due to randoms. For iteration *k*:

$$\begin{array}{c} q_{l}^{\left(k\right)}=\sum_{w}A_{lw}f_{w}^{\left(k\right)}+r_{l}^{\text{mth}}, \end{array}$$ (10)

where $r_{l}^{\text{mth}}$ is the expected number of random counts in *l* estimated by the method mth. Images of the IQ phantom were reconstructed and the following regions of interest (RoIs) were defined: a *hot* RoI and a *cold* RoI were centred inside the cavity with the highest activity concentration, and the empty cavity, respectively. A *warm* RoI was defined in the homogeneous region of the phantom. In the following, the hot, cold and warm RoIs will be indicated by the subscripts *h*, *c* and *w* respectively. Standard FoMs were calculated:

- *Contrast (C)* between RoI *a* and its background *b* is defined as:
  $$\begin{array}{c} C_{\left(a/b\right)}=\frac{\mu_{r,a}}{\mu_{r,b}}-1, \end{array}$$ (11)
  where *μ*_*r*,*α*_ represents the mean value of the reconstructed intensity in RoI *α*. Ideally, *C*_(*h*/*w*)_ = 3.
- *Contrast Recovery Coefficient (CRC)*: It is defined as
  $$\begin{array}{c} CRC_{\left(a/b\right)}=\frac{\frac{\mu_{r,a}}{\mu_{r,b}}-1}{\frac{\mu_{t,a}}{\mu_{t,b}}-1}, \end{array}$$ (12)
  where *μ*_*t*,*α*_ represents the true mean intensity value in RoI *α*. Ideally, *CRC*_(*h*/*w*)_ = 1.
- *Spill–over ratio (SOR)*: It is defined for the cold RoI as
  $$\begin{array}{c} SOR=\frac{\mu_{r,c}}{\mu_{r,w}}. \end{array}$$ (13)
  Ideally, *SOR* = 0.
- *Image Roughness (IR)*: The IR in a RoI measures the pixel–to–pixel variability and can be calculated for a single realization. Image Roughness is the image noise perceived when viewing an individual image [39]
  $$\begin{array}{c} IR_{a}=\frac{\sqrt{\frac{1}{V-1}\sum_{v\in a}{\left(f_{v,a}^{r}-\mu_{r,a}\right)}^{2}}}{\mu_{r,a}}, \end{array}$$ (14)
  where $f_{v,\alpha }^{r}$ is the reconstructed intensity in voxel *v* of RoI *α* and *V* is the total number of voxels in RoI *α*.
- *Regional Bias (RB)* in a RoI *a* is defined as:
  $$\begin{array}{c} RB_{a}=\frac{\mu_{r,a}}{\mu_{t,a}}-1 \end{array}$$ (15)
  Ideally, for any RoI *α*, *RB*_*α*_ = 0.

The total activity of the phantom, 1.5 mCi (55.5 MBq), has been selected following the usual strategy of working at the NECR peak. Incidentally, NECR peaks for DW and SP are achieved at the same activity, see Results.

All the FoMs have been calculated up to 100 iterations.

Preliminary results revealed that, for a given activity, the statistical level affected differently the estimation methods. To study this effect, we prepared two data sets with different statistics: a short set obtained by acquiring data during 1 s, and a long set obtained by acquiring data during 10 s. Both at the *same* activity, 1.5 mCi (55.5 MBq). Since the long set corresponds to a higher statistics scenario than the short set, the two sets will be referred in the following as the *low* and *high* statistics sets. The names reflect only the fact that one has lower statistics than the other. The average number of emitted events inside any voxel of the warm region is ≈ 2 ⋅ 10^3^ (*low* set), and 2 ⋅ 10^4^ (*high*). A third set of 100 s was also simulated but the results were similar to those of 10 s (and thus not shown here).

As shown later, the low statistics scenarios are more challenging. Therefore, for the MOBY phantom, the total activity and simulation time have been selected to generate a low-statistics set of data: 1.5 mCi (55.5 MBq) and 1 s, respectively. The Correlation Coefficient (CC) between the reconstructed image and the original activity distribution has been calculated:

$$\begin{array}{c} CC=\frac{{\vec{f}}^{r}\cdot {\vec{f}}^{t}}{|{\vec{f}}^{r}\left|\right|{\vec{f}}^{t}|}=\frac{\sum_{v}f_{v}^{r}f_{v}^{t}}{\sqrt{\sum_{v}f_{v}^{r}f_{v}^{r}}\;\;\sqrt{\sum_{v}f_{v}^{t}f_{v}^{t}}}, \end{array}$$ (16)

where $f_{v}^{t}$ and $f_{v}^{r}$ stand for the true and reconstructed intensities in voxel *v*. The values of the CC were used to obtain an objective estimation of the number of iterations at which images may be compared.

## Results

### Assessment of the estimators direct output

**Fig 3:**
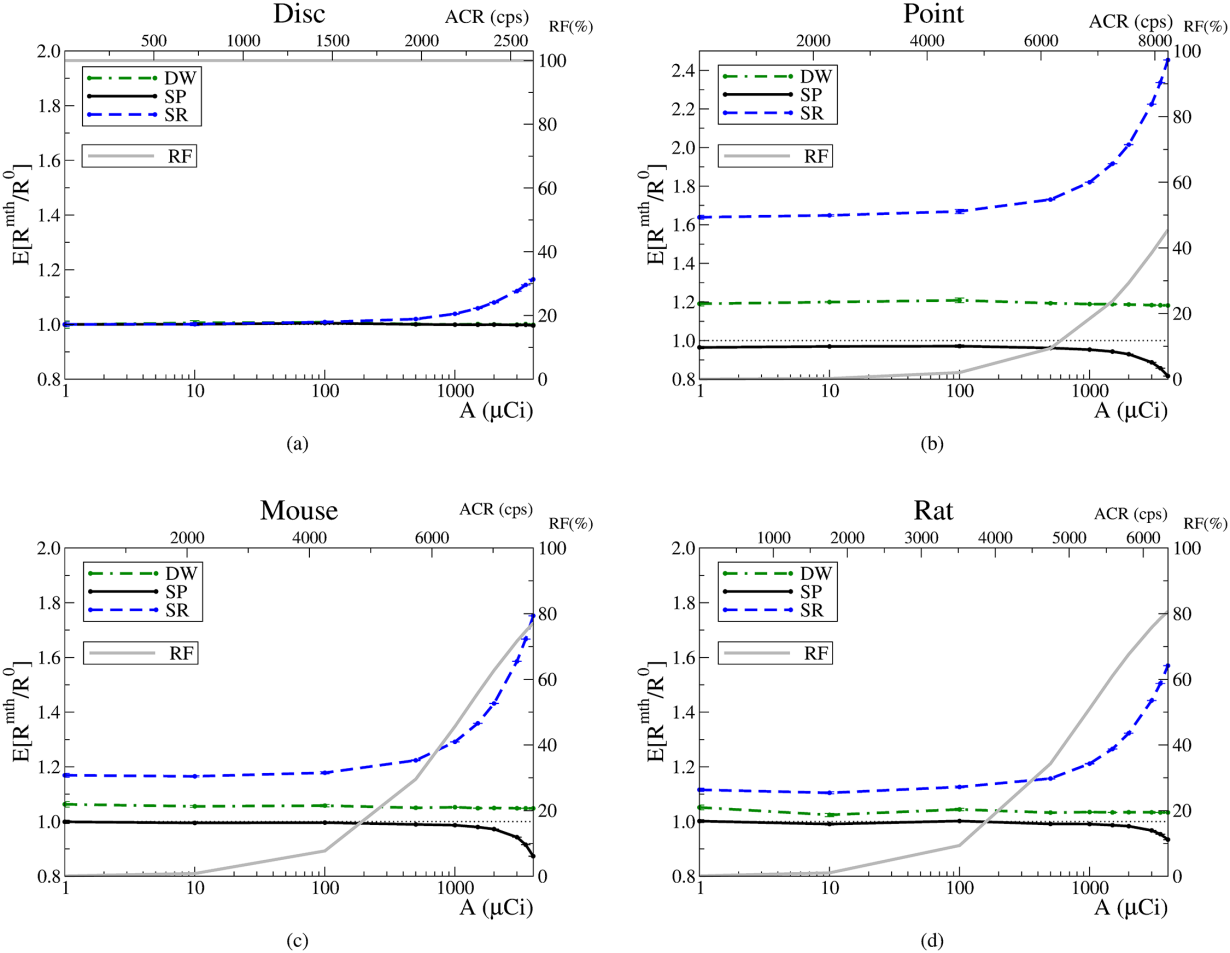
Estimated random rates and random fractions. Error bars correspond to one standard deviation. Estimates provided by the ideal method are represented as a horizontal dotted line; SP: solid line; SR: dashed line, and DW: dotted–solid line. The average singles count rate (ACR) per detector is shown in the upper *x* axis. The RF is represented by a grey solid line.

**Fig 4:**
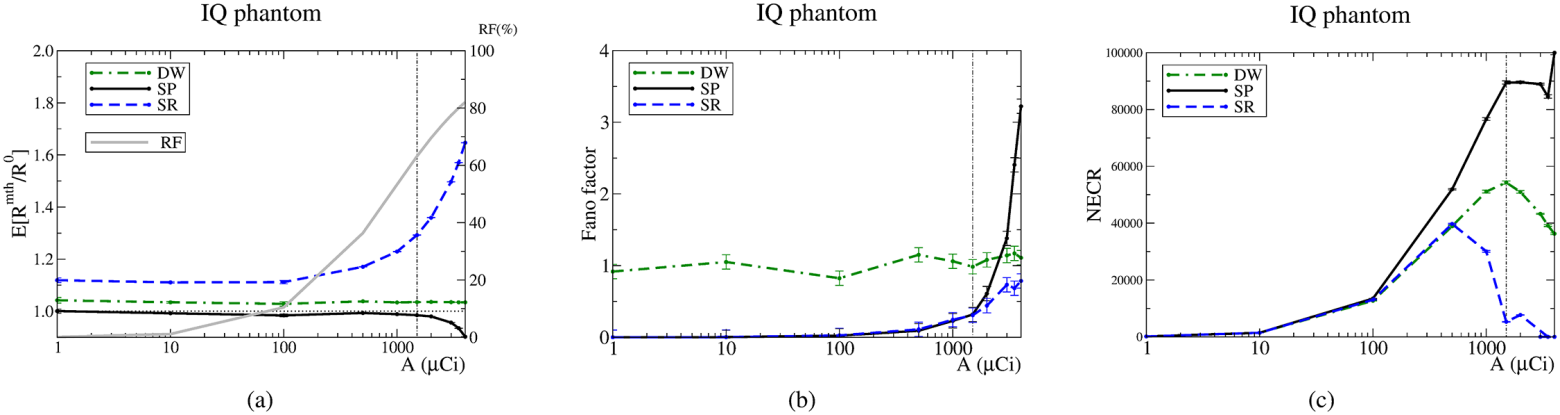
Estimations provided by the methods, the Fano factors and NECR for the IQ phantom. The vertical line corresponds to the NECR value used. SP: solid line; SR: dashed line, and DW: dotted–solid line. The RF is represented by a grey solid line.

The variation of *E*[*R*^mth^/*R*^0^] with the activity is shown in Figs 3 and 4a. The dependency on the average singles count rate per detector, ACR, is also shown in the upper *x* axis. Upon visual examination, two regimes can be distinguished which we refer to as *low* and *high* activity regimes. The limit between the two is around 1 mCi (37 MBq). Above this activity, the SR estimation quickly degrades, and the SP method starts to underestimate the correct value. Note that the DW performance does not change when passing from one regime to the other.

Let us focus on the low activity regime. It is worth to stress that for the disc phantom the three methods provide an accurate estimation of the random rate, Fig 3a. For the other phantoms, the two conventional methods, SR and DW, systematically overestimate the correct value. The deviations with respect to the correct value prove to be constant. The particular value of the overestimation depends on the phantom and is more pronounced for the point source. In contrast, it must be emphasized that the SP estimation is compatible with the correct value for all phantoms except the point source. The largest disagreement for SP occurs at 1 mCi (37 MBq), but the underestimation amounts only to -4.7% (DW overestimates 19% and SR 82%).

For the high activity regime, SR and SP estimations significantly degrade. The overestimation caused by SR increases notably for all phantoms. For SP, the degradation comes as an underestimation that becomes more severe as the activity increases. However, SP still provides the best estimation available in this regime, except at the highest investigated activity for the Rat and Mouse cases. In contrast with SR and SP, the overestimation associated to DW remains unchanged.

The values obtained for the Fano factors, Fig 4b, agree with the values found in the literature. For the SR method, the results confirm that neglecting its variance is an excellent approximation up to high activities. However, the results also reveal that the approximation ceases to be correct for high activities. On the contrary, for the DW method, the conventional approximation holds for all activities. Although for the SP method the variance increases as the activity is increased, it is worth to emphasize that, for activities below approximately 1 mCi (37 MBq), its Fano factor is negligible and equal to that of the SR method. Therefore, except for high activities, the SP method presents a negligible variance while providing accurate estimates.

Regarding the NECR, Fig 4c, for activities below 0.1 mCi (3.7 MBq), the three estimation methods provide the same NECR. In contrast, for higher activities the SP method always provides higher NECR values than SR and DW. The activity at which the NECR peak is reached is lower for SR than for DW and SP. Incidentally, for the latter two the peak is reached at about 1.5 mCi (55.5 MBq), being the highest NECR value the one achieved by the SP method. The anomalous increment at the last point of the NECR for the SP method is due to the fact that SP becomes a biased estimator for activities above 2 mCi (74 MBq). For these activities, the systematic SP underestimation tends to artificially enhance the NECR. To confirm this, we have estimated the bias from the results shown in Fig 4a and recomputed the NECR by taking into account the bias. Then, the peak in the last point disappears and the NECR behaves as expected, i.e. beyond the NECR peak the NECR decreases as the activity increases.

In summary, for all the FoMs analysed in this section, the SP method performs best. For a wide range of activities and source distributions, SP provides an accurate estimation while keeping a low variance.

### Image Quality assessment

**Fig 5:**
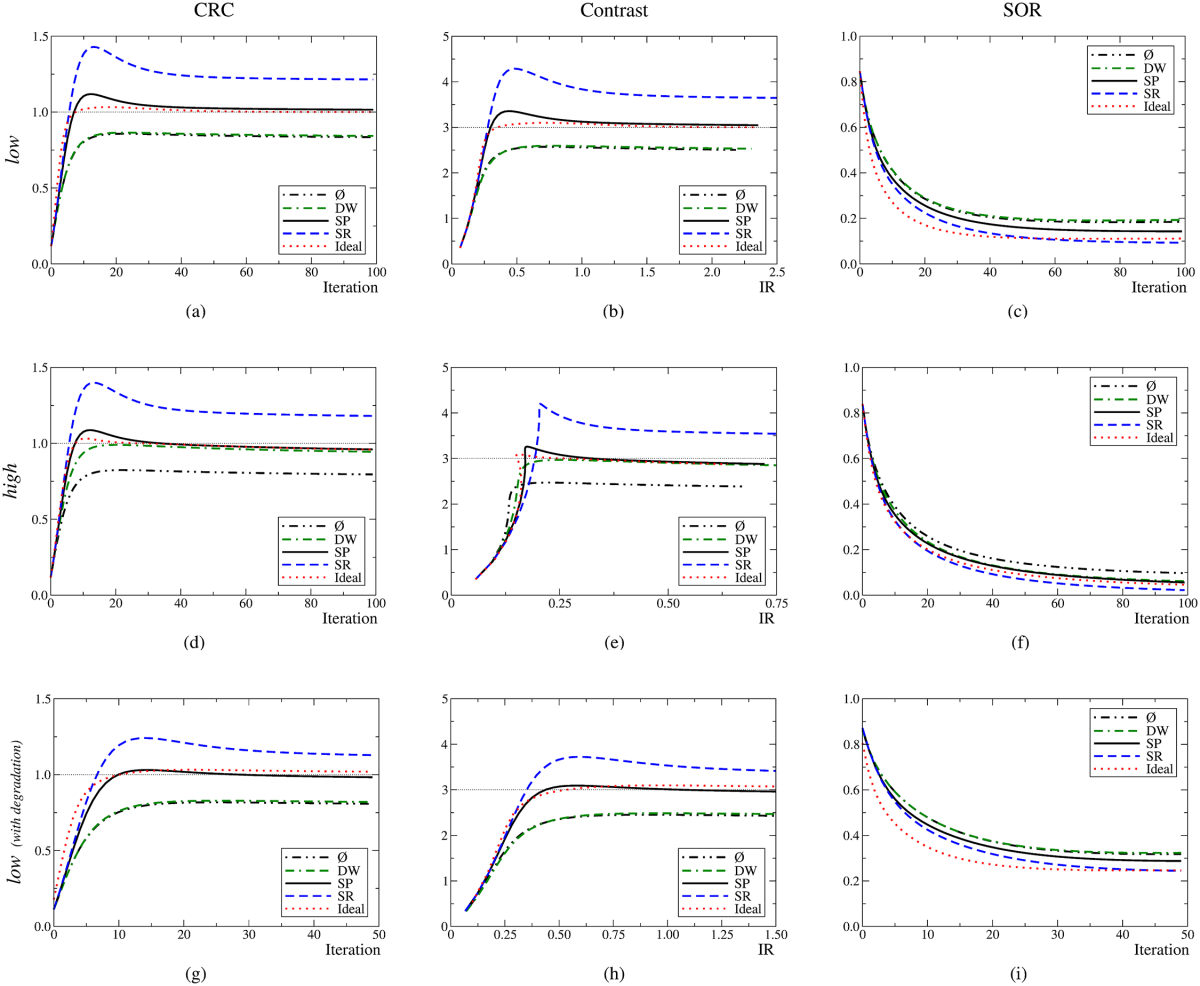
First row: low set; second row: high set. Third row: low set including the degradation effects. Ideal value: horizontal line; ideal method: dotted line; SP: solid line; SR: dashed line; DW: dotted–solid line, and no compensation applied (represented by the symbol ⌀): dot–dot–dash.

The graphs corresponding to the CRC, C *vs* IR and SOR are shown in Fig 5. The first and second rows correspond to the *low* (1 s) and *high* (10 s) statistics scenarios, respectively. The graphs show that convergence is achieved around 40 iterations. The graphs obtained for the simulation set of 100 s (not shown) display the same trends than those obtained for the 10 s except for the fact the the maximum image noise value, IR, is 0.25 instead of 0.75.

Let us focus on the low statistics scenario. For all the FoMs, the values obtained when using the DW method are very similar than those obtained without randoms compensation. In contrast, the SP method performs similarly to the ideal one. Particularly, the SP method achieves the correct value for the contrast, 3, while the SR method converges to approximately 3.6 and the DW to about 2.5. The SOR converges more slowly. The SP method outperforms the DW method, and the SOR values provided by the SR approach are closer to the ideal value of zero.

For the high statistics scenario, the main outcome is that the DW method provides similar results than the SP and ideal methods. The SR method keeps overestimating the contrast, +17%. As expected, no compensation for randoms translated into low-contrast images, −20%. On the contrary, the DW, SP and ideal methods provide values close to the correct value, 3; $C_{h/w}^{\text{DW}}=2.92$ and $C_{h/w}^{\text{SP}}=C_{h/w}^{\text{0}}=2.98$. The growth pattern of the image noise qualitatively changes with respect to the low statistics scenario, and the higher statistics translates into an image noise reduction of about 70% at convergence. For the SOR, the DW, SP and ideal methods yield similar outputs while the SR method provides the best performance.

The graphs on the bottom row of Fig 5 correspond to the CRC, C *vs* IR and SOR when all the degradation effects (attenuation, scatter, acollinearity, positron range and dead–time) are taken into account. For this case, the randoms fraction was 63% and the scatter fraction was 5%. Qualitatively, the same trends emerge as when no other degradation effects are included.

**Fig 6:**
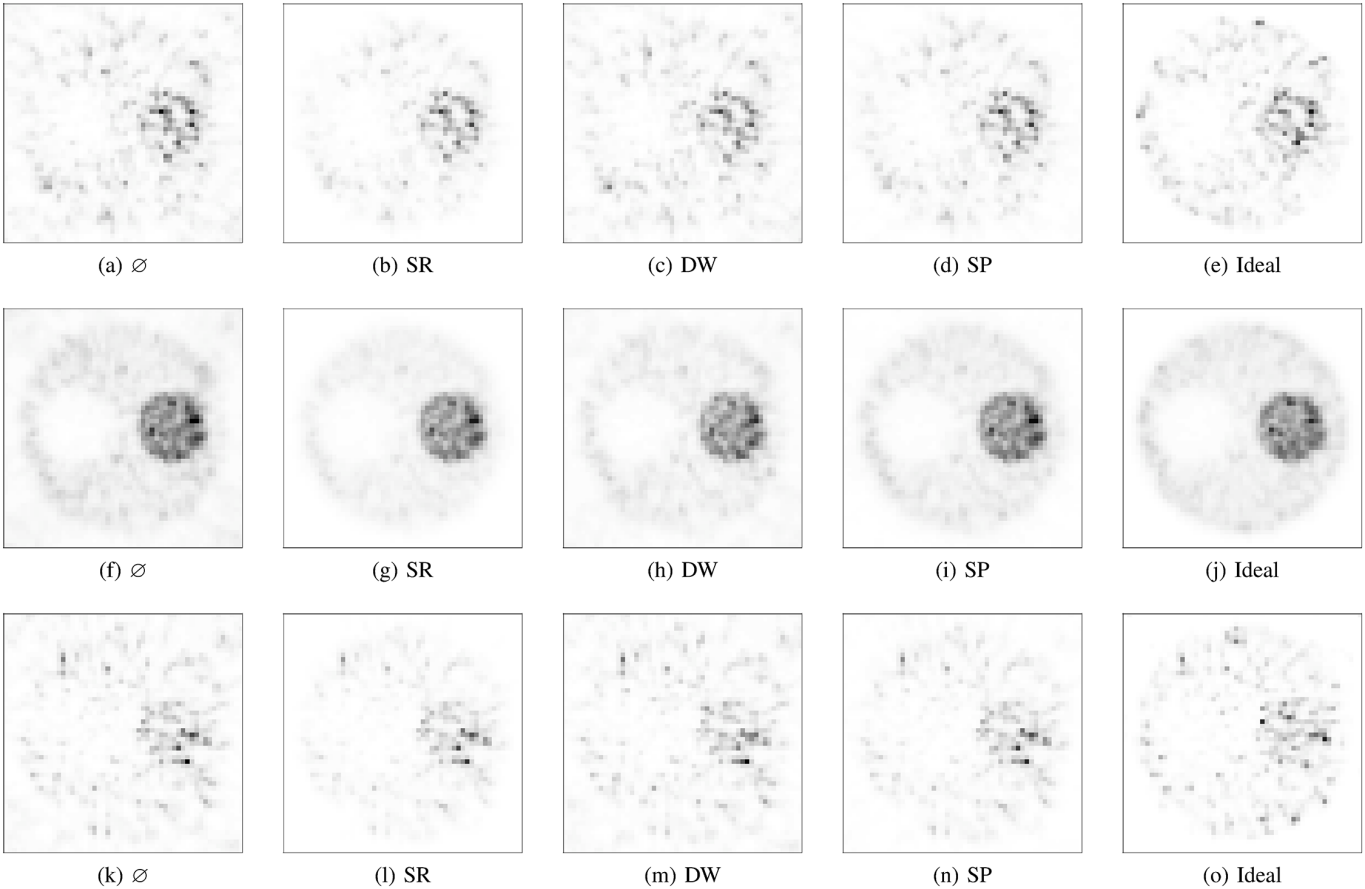
Transaxial central slice of the IQ phantom at 40 iterations. First row: low set; second row: high set; third row: images with all the degradation effects. The images in the same row share the same colour scale, which has been normalized to the pixel with the maximum intensity.

The corresponding images are very similar (Fig 6) and the trends followed by the FoMs can be visually assessed. A new issue is also observed: the DW method generates images with a significant external background so that the outer boundary of the phantom becomes blurred. In contrast, the SP method generates a low external background, and SR causes the best visual impression (not counting the ideal method).

Visually the images appear noisy, an aspect that is partially covered by the IR FoM. To complete the quantification of image quality, we have also computed the regional bias, RB, by using as a reference the bias–free ideal method. The results are shown in Table 1

**Table 1:** Regional bias computed for the hot and warm regions.

	Warm		Hot	
	low	high	low	high
⌀	+20%	+21%	+6.3%	+4.3%
**SR**	-18%	-18%	-3.2%	-5.0%
**DW**	+9.7%	-6.8%	-2.0%	-8.6%
**SP**	-1.6%	-0.54%	+0.66%	-1.1%

**Fig 7:**
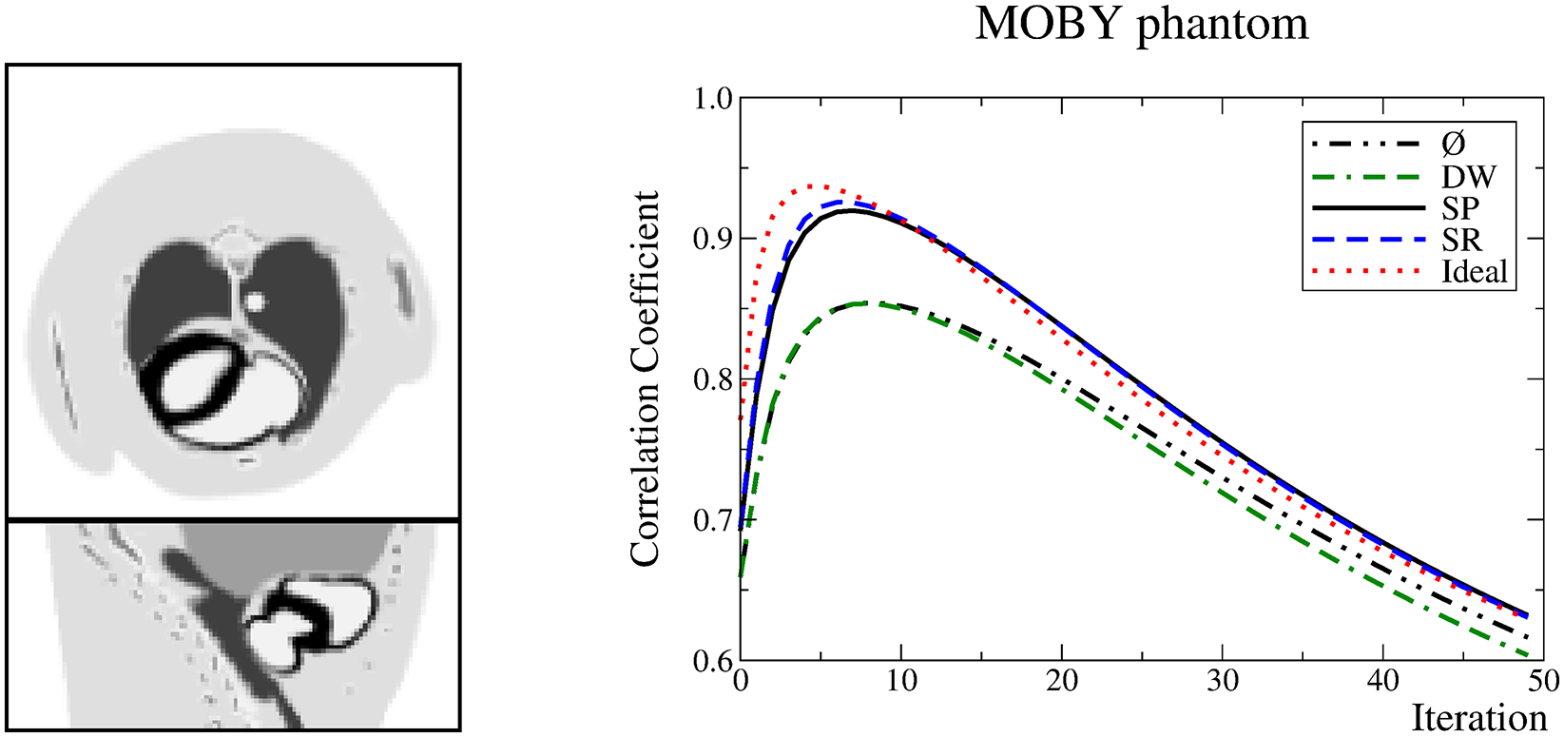
Left: central transaxial and coronal slices of the simulated MOBY phantom. Right: graph of the CC achieved by the methods.

Regarding the CC for the MOBY phantom, Fig 7, the DW method performs as if no method were used. The SP and SR methods perform very similarly and achieve a higher CC than the DW method. The best value for the CC is obtained in the seventh iteration. Visual inspection revealed that the previously observed trends were reproduced. Images are not shown since there are no relevant information. In particular, the SR method produced a sharper external boundary, followed by SP, and then DW. Again, the contrast was artificially enhanced by SR.

## Discussion

### Assessment of the estimators direct output

The SR method systematically overestimates the correct value of the randoms rate, Figs 3 and 4a. This outcome can be understood by reckoning that SR is based on the singles rates, *S*_*i*_, which includes the contributions not only from the uncorrelated singles but also from the correlated singles. Therefore, *S*_*i*_ ≥ *λ*_*i*_, which implies that $R_{ij}^{\text{SR}}\geq 2\tau \lambda_{i}\lambda_{j}$. The equality holds when no correlated singles are present in the data, *S*_*i*_ = *λ*_*i*_. In fact, this situation happens for the disc phantom; therefore, the SR estimation should be accurate (when pile–up can be neglected, see below). Actually, this is in agreement with the results shown in Fig 3a.

A similar reasoning applies to the DW method. By obtaining coincidences between the original (undelayed) data stream and the delayed stream, the correlations between the events are broken. However, nothing prevents the DW method to include the additional contribution from the correlated singles. This extra contribution translates into overestimations than can be seen in Figs 3 and 4a. An accurate DW estimation would thus require to remove the correlated singles from the streams. For the disc, the DW estimation is accurate because no correlated singles are present, while for the rest of the phantoms the DW estimation degrades due to the correlated singles present in the data. The higher the contribution of correlated singles, the larger the overestimation.

The results presented in Figs 3 and 4a show that the performance of the SR method is particularly sensitive to the activity and presents an anomalous overestimation for the high activity regime. The nature of this phenomenon can be understood by inspecting the graph corresponding to the disc, Fig 3a. This phantom provides no correlated singles, i.e. *S*_*i*_ = *λ*_*i*_. Therefore, all three methods should provide the correct value. Although this is the case in the low activity regime, the SR estimator fails to provide the correct value for the high activity regime. To understand this failure, consider that in this situation, Eq (2) can be written as $R_{ij}^{0}\approx R_{ij}^{\text{SR}}\;e^{-2\Lambda \tau }$. The latter formula can be used to quantify the SR overestimation: *E*[*R*^SR^/*R*^0^] ≈ *e*^2 Λ(*A*)*τ*^, where we have emphasized that Λ depends on the underlying activity. Hence, the disagreement at high activities for the disc arises from the fact that SR neglects pile–up. We have computed (not shown) that the value obtained for Λ from the simulation accounts for the deviation of SR estimation in the high activity regimes. As a consequence, we speculate that the reason behind the existence of two regimes is deeply related to pile–up. Actually, the two regimes would correspond to two scenarios: one in which the pile–up can be ignored and another one in which it has to be taken into account.

In contrast to SR, the SP method does take into account the pile–up. Hence, it is able to provide a correct estimation of the randoms rate in any regime. Essentially, the SP method estimates the formula in Eq (2) from measured data. This estimation includes the value of the pile–up compensating factor, *e*^ − 2Λ*τ*^. For the disc, where no correlated singles are present, SP is able to properly estimate the factor. However, for the rest of the phantoms, the compensation for pile–up is not accurate enough, which explains the small underestimation (-6%) for the highest activity (3 mCi).

Remarkably, the DW method is insensitive to the working regime and its estimation does not further degrade when the activity is increased. One possible reason is that, during the sorting process, the pile–up equally affects the regular and the delayed streams. Yet, the DW begins to be competitive with SP only at very high activities (≳ 3 mCi). Therefore, a situation in which the DW method outperforms the SP method implies an scenario in which pile–up effects are very important. In that scenario, the corresponding count–rates losses would be so high that a reduction of the activity would be advisable.

Regarding the variance of the estimations, the fact that the SR and DW exhibit different values of the Fano factors, *F*^SR^ = 0 and *F*^DW^ = 1, is because each method is based on a completely different approach. But, while the former is based on singles (high statistics, low variance), the latter is based on coincidences (lower statistics, higher variance). A particularly advantageous feature of the SP method is that, although it is based on singles as well as on coincidences, the resulting variance is similar to that of the SR method. This outcome was somehow expected because the SP method is actually based on counting *effective* singles Eq (6): ${\bar{S}}_{k}\equiv S_{k}-P_{k}\;e^{\tau (\lambda +S)}$. For not very high activities, the exponential factor becomes the unity and since the singles are more abundant than the prompts, then ${\bar{S}}_{k}\approx S_{k}$.

To analyse the NECR results, it is relevant to mention that when the NECR is used in any study, it is implicitly assumed that the randoms estimation method used is unbiased, i.e. the trues can be estimated as *P* − *R*^mth^. However, our results reveal that the conventional randoms estimation methods are biased. As a consequence, *P* − *R*^mth^ constitutes a biased estimator. Incidentally, the NECR penalizes methods that overestimate the randoms because the trues are underestimated. On the contrary, the NECR is artificially improved for methods that underestimate randoms, as the trues are thus overestimated. Hence, in our work the use of the NECR for comparison purposes is justified except for the SP method at very high activities, ≳ 2 mCi, where the related underestimation becomes non–negligible.

In terms of the NECR, the SP method is always better than the conventional methods, Fig 4c. To understand this, consider that, although the SR method presents a very low variance (comparable to that of the SP method), Fig 4b, the NECR values are worse for the former because of the strong overestimation, Fig 4a. The NECR values for the DW method are also worse than those of the SP method because the DW method not only overestimates the correct value, Fig 4a, but also its variance is higher than that of the SP method, Fig 4b.

### Image quality enhancement

The size and placement of the voxels within each RoI were defined to avoid border and spatial–resolution related effects, so that the voxels were completely contained within homogeneous regions.

**Fig 8:**
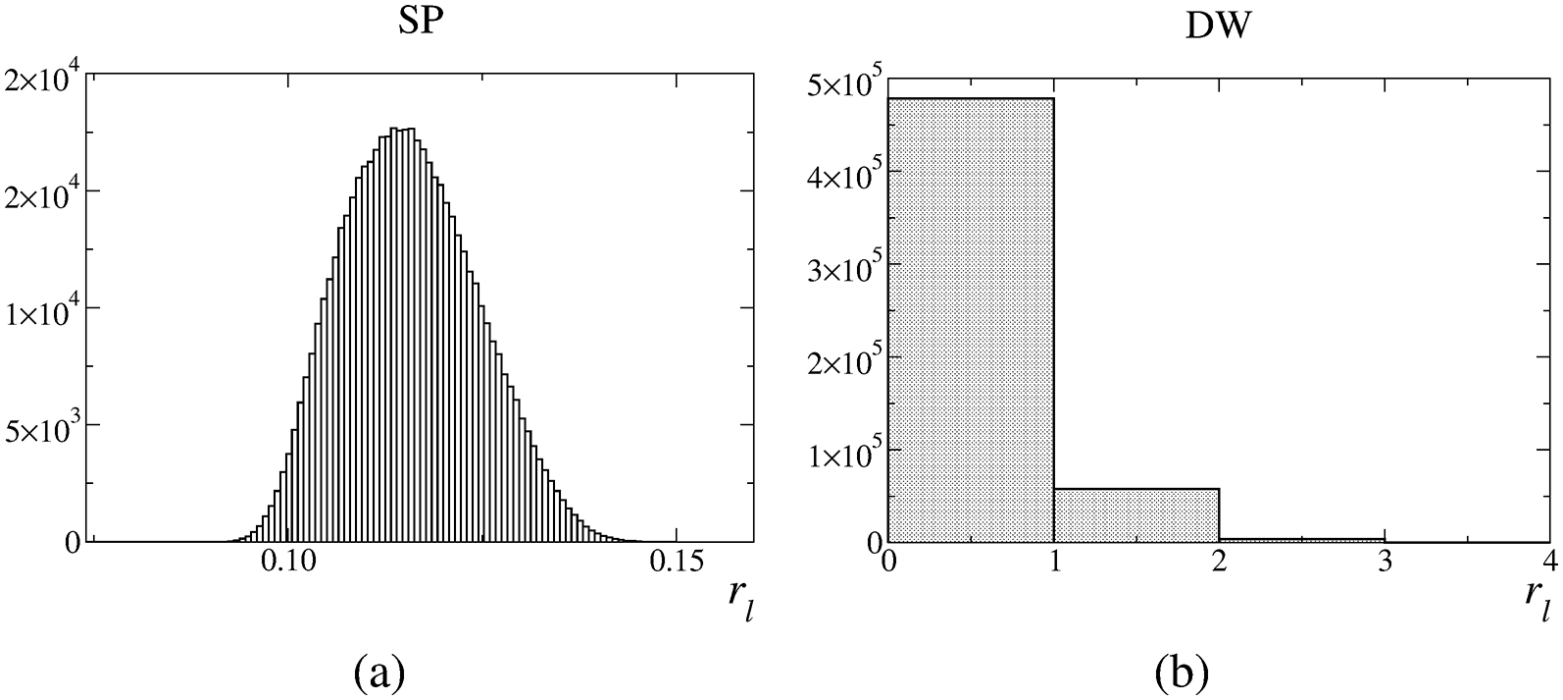
Histograms of the estimated values of $r_{l}^{\text{mth}}$.

In general, the FoMs reveal that improved estimates translate into improved image quality. In the end, the systematic overestimations of the SR method produce images of lower quality. This method artificially enhances the contrast. The SP method provides accurate FoMs, whose values are very similar to those obtained by using the ideal method. The overestimations of the DW method do not affect significantly the images. In general, the image quality is similar to that obtained with the SP and ideal methods. However, for low statistics, DW images do not follow the same trend as the SR images. Interestingly, the DW overestimation translates into a reduced contrast. Moreover, unlike the SR method, the DW method appears to be very sensitive to the statistical quality of the data. While for the low statistics scenario using the DW estimate into the image reconstruction is equivalent to not compensating for randoms, for the high statistics scenario, using the DW estimate produces results very similar to using the SP or the ideal estimates. To clarify this aspect, we have calculated the histograms of the estimated number of randoms in each LoR, $r_{l}^{\text{mth}}$, Fig 8. SP produces values of *r*_*l*_ which are distributed around 0.12. The SP method is able to provide real values for *r*_*l*_. Consequently, it can offer accurate estimations for non–integer values. However, by definition, the DW method can only provide integer values. For the present situation, the best integer value that can be obtained is 0, which is actually what DW mainly provides. This explains why the DW method equals not using any compensation method. These results suggest that, for low statistics, the inability of the DW method to adapt to non-integer small values of *r*_*l*_ results in a degradation of image quality comparable to that obtained when no randoms compensation is applied. For these scenarios, the DW method would require to additionally apply variance reduction techniques [40, 41]. Scenarios with noisy images due to low statistics are not uncommon, e.g. dynamic studies with narrow time frames (i.e., small number of events per frame) [42].

Regarding the external background outside the phantom, accurate estimates for this region are not so relevant as for the inner part of the phantom. In this sense, although the SR method produces a lower background, SP-based images are better from a quantitative point of view (and show acceptable external background levels). In terms of regional bias, the results shown in Table 1 further confirm that for all the studied scenarios the SP is the best method followed by the DW, SR and ⌀. Consistently, the CC as well as the images obtained for the MOBY agree with the previous results.

## Conclusions

SP, a novel method for randoms rate estimation, has been thoroughly assessed. Based on the well–known SR method, the SP estimator improves SR in two aspects. First, SP takes into account that the randoms are predominantly made up of uncorrelated singles. While the SR estimator uses all the singles measured by each detector, SP estimates the number of uncorrelated singles present in the data and uses them to accurately estimate the randoms rate. Second, the SP method includes the pile–up effects, i.e. it takes into account the probability of finding more than one single inside the TCW (the SR method ignores this).

The SP estimation formula can be written in the same mathematical form as the SR one, $R_{ij}^{\text{SP}}=2\bar{\tau }{\bar{S}}_{i}{\bar{S}}_{j}$, where $\bar{\tau }$ and ${\bar{S}}_{i}$ are the effective TCW and singles rate, respectively. Moreover, SP does not require any additional measurement.

Monte–Carlo simulations allowed us to compare the randoms rate estimates provided by different methods to the actual randoms rate present in the data. We have compared the proposed SP method to the two most commonly used techniques: the SR and the DW methods. The performance of these three techniques has been assessed at two levels: (1) comparison of the randoms rate predicted by each method, and (2) comparison of the reconstructed images compensated for randoms. At the direct output level, the results show that, in general, SP outperforms other methods. While the SR and DW systematically overestimate the true randoms rate, the SP method is able to accurately provide the correct value. At the level of reconstructed images, the accuracy of the SP method translates into improved image quality. The FoMs reveal the better performance of the SP method. The FoM values related to the SP method are similar to those that would have been obtained by using ideal randoms estimates. Particularly, for low statistics scenarios, the SP method is the only method able to produce the proper contrast. For high statistics scenarios, the SP and DW methods yield similar FoM values. Visual inspection of the IQ and MOBY images (not shown) reveal an external background that makes difficult to delineate the phantom boundary. Although the SR method generates a lower background than SP (and SP less than the DW), the contrast of the SP-based is correct while the SR method results in overestimated contrast. Up to this point, the results reported have been based on simulations that ignored several degradation effects in order to focus on randoms. Nevertheless, simulations including these effects were also performed for the IQ phantom. The results show that the aforementioned trends are qualitatively preserved.

In summary, the SP method is a better estimator than the conventional SR method, which translates into better image quality. The SP estimation is of the same level of complexity than the SR one and does not require any extra measurement. For any system incorporating the SR method as a random compensation technique, the replacement of SR by SP would be simple. In general, the DW and the SP methods offer similar results except for low statistics scenarios where the DW needs to be complemented with variance reduction techniques. For these scenarios, the SP method straightforwardly provides reliable and accurate estimations.

## Appendix

### Derivation of the SP estimation formula

Here we offer a simplified derivation of Eq (3). The prompts rate in the LoR defined by the detectors *i* and *j* can be estimated as the rate of correlated singles plus the rate of uncorrelated singles. By using Eq (2), it follows:

$$\begin{array}{c} P_{ij}=\left(\rho_{ij}+2\tau \lambda_{i}\lambda_{j}\right)e^{-2\Lambda \tau }. \end{array}$$ (17)

The demonstration that the pile–up factor, *e*^−2Λ*τ*^, also affects the correlated singles rate, *ρ*_*ij*_, can be straightforwardly derived by following similar steps to those described in [10]. The singles rate in the detector *i* can also be estimated as the sum of the two contributions:

$$\begin{array}{c} S_{i}=\rho_{i}+\lambda_{i}, \end{array}$$ (18)

where *ρ*_*i*_ = ∑_*j*_ *ρ*_*ij*_. Note that neither *τ* nor *e*^−2Λ*τ*^ appear in Eq (18). (To count singles there is no need to extract any coincidence.) Upon summing over all indices in the previous equations we obtain:

$$\begin{array}{ccc} P & = & \left(\rho +2\tau \lambda^{2}\right)\;e^{-2\Lambda \tau } \end{array}$$ (19)

$$\begin{array}{ccc} S & = & \rho +\lambda \end{array}$$ (20)

$$\begin{array}{ccc} \Lambda & = & \lambda +\rho /2 \end{array}$$ (21)

where we have added the third equation for completeness. By combining these expressions, *λ* can be found as the solution to

$$\begin{array}{c} 2\tau \lambda^{2}-\lambda +S-P\;e^{(\lambda +S)\tau }=0, \end{array}$$ (22)

and Λ = (*S*+*λ*)/2. The estimation of the prompts rate in detector *i* can be found upon application of the previous results to Eq (17):

$$\begin{array}{c} P_{i}=\left(\rho_{i}+2\tau \lambda \lambda_{i}\right)\;e^{-(S+\lambda )\tau }. \end{array}$$ (23)

Eqs (23) and (18) provide an estimation for *λ*_*i*_

$$\begin{array}{c} \lambda_{i}=\frac{S_{i}-P_{i}\;e^{(S+\lambda )\tau }}{1-2\lambda \tau }. \end{array}$$ (24)

Finally, the original equation Eq (2) can be expressed as

$$\begin{array}{c} R_{ij}^{\text{SP}}=\frac{2\tau e^{-(\lambda +S)\tau }}{{\left(1-2\lambda \tau \right)}^{2}}\left(S_{i}-e^{(\lambda +S)\tau }\;P_{i}\right)\left(S_{j}-e^{(\lambda +S)\tau }\;P_{j}\right). \end{array}$$ (25)

See also [28] for further results of the model.

## Supporting Information

S1 Data Data used the graphs (xmgrace). (ZIP)
